# Infrared Thermal Imaging of Patients With Acute Upper Respiratory Tract Infection: Mixed Methods Analysis

**DOI:** 10.2196/22524

**Published:** 2021-08-19

**Authors:** Zuopeng Zhang, ZanFeng Cao, Fangge Deng, Zhanzheng Yang, Sige Ma, Qianting Guan, Rong Liu, Zhuosen He

**Affiliations:** 1 Emergency Department The First Affiliated Hospital of Guangzhou Medical University Guangzhou China; 2 State Key Laboratory of Respiratory Diseases The First Affiliated Hospital of Guangzhou Medical University Guangzhou China; 3 The First Clinical College Guangzhou Medical University Guangzhou China

**Keywords:** acute upper respiratory tract, infrared thermography, qualitative study, quantitative study

## Abstract

**Background:**

Upper respiratory tract infection is a common disease of the respiratory system. Its incidence is very high, and it can even cause pandemics. Infrared thermal imaging (IRTI) can provide an objective and quantifiable reference for the visual diagnosis of people with acute respiratory tract infection, and it can function as an effective indicator of clinical diagnosis.

**Objective:**

The aims of this study are to observe and analyze the infrared expression location and characteristics of patients with acute upper respiratory tract infection through IRTI technology and to clearly express the quantification of temperature, analyze the role of IRTI in acute upper respiratory tract diagnostic research, and understand the impact of IRTI in qualitative and quantitative research.

**Methods:**

From December 2018 to February 2019, 154 patients with acute upper respiratory tract infection were randomly selected from the emergency department of the First Affiliated Hospital of Guangzhou Medical University. Among these patients, 73 were men and 81 were women. The subjects were divided into two groups according to the presence of fever, namely, fever and nonfever groups. Qualitative and quantitative analyses of the infrared thermal images were performed to compare the results before and after application of the technology.

**Results:**

Using the method described in this paper, through the analysis of experimental data, we elucidated the role of IRTI in the diagnosis of acute upper respiratory tract infection, and we found that qualitative and quantitative IRTI analyses play important roles. Through the combination of theory and experimental data, the IRTI analysis showed good results in identifying acute upper respiratory tract infection.

**Conclusions:**

IRTI technology plays an important role in identifying the infrared expression location and characteristics of patients with acute upper respiratory tract infection as well as in the quantification of clear expression of body temperature, and it provides an objective and quantifiable reference basis for elucidating the pathogenesis of these patients.

## Introduction

### Background

Acute upper respiratory infection is common in winter and spring. It can be spread through food droplets containing influenza virus or the use of virus-contaminated robotic arms and other tools [1,2]. These viruses may also cause local or large-scale global climate change and pandemics [3,4]. Because the antigen on the surface of this subtype of human virus can readily directly undergo immune mutation, new immune subtypes are generated, and there is no possibility of direct crossover between different novel subtypes for immunization. Therefore, the disease can not only occur repeatedly within one year in the same person but can also cause widespread epidemics in a few years [5,6].

Far-infrared thermal imaging is a noninvasive, nontoxic, and objective temperature measurement method that can provide information on changes in the function of the parasympathetic nervous system [7]. By collecting the far-infrared radiation heat from the human body, determining an intuitive temperature, processing a color map through a computer, and using different colors to represent different human body surface temperature distributions, the temperature distribution changes of the human body can be accurately measured according to the difference between normal and abnormal infrared radiation [8]. Based on the scope and location of the focus, this is a functional image that can reflect the metabolism of the body; however, the observation of subtle changes in tissue structure using this method is not as accurate as that using computed tomography or magnetic resonance imaging [9,10]. Temperature is an important indicator that can reflect the pathophysiological state of the human body. The skin temperature of the normal human body is basically symmetrical from the limbs to the head and face [11]. When people contract diseases, the metabolism of local tissues and cells changes first, prior to changes in human function or morphology. Moreover, before anatomical structure changes, molecular biology changes occur in lesions and their surrounding tissues, which will further change the normal temperature of the lesion. The spatial distribution and gradient change of temperature will further reflect the specific scope of the disease and reveal the nature of the occurrence and development of the disease [12]. Far-infrared thermal imaging technology is based on this principle; through a series of computer analyses and the use of image processing technology to collect infrared information, thermal images can be formed using different colors to display the temperature distribution of the human surface [13].

### Infrared Thermal Imaging

Infrared thermal imaging (IRTI) temperature measurement technology was developed on the basis of the development of an infrared focal plane array. An IRTI temperature measurement system is a 2D thermal imaging temperature measurement device [14]. The system detects and displays the distribution of infrared radiation energy density based on between the target and the environment according to the gray value of the gray image. At present, there are two main types of infrared detectors: photon detectors and thermal detectors. Photon detectors include photoelectric detectors, photoconductive detectors, and quantum detectors. The working performance of a photon radiation detector is only very good when the ambient temperature is very low. Thermal radiation detectors mainly include thermocouples, thermoelectric reactors, thermal release relays and other detectors, as well as various thermal radiation heaters [15]. Thermal radiation detectors have relatively broad control requirements for the temperature of specific working environments. These detectors can usually function normally at room temperature. Infrared detectors that can function at room temperature are often called uncooled infrared detectors because they do not require refrigeration equipment [16]. Pyroelectric detectors and thermal radiation calorimeters are two widely used instruments in thermal infrared detectors. Thermal radiation detectors are more mature than heaters for the measurement of electrical radiation. Infrared camera technology is an important product of the mature application and development of infrared focus plane imaging technology [17]. As a new type of automatic gas temperature measurement and control technology, one of the main features of infrared temperature measurement is the noncontact automatic measurement of gas temperature. In addition, compared with traditional temperature measurement methods, it has two advantages of low automatic temperature measurement and high accuracy. Compared with traditional infrared contact thermal temperature measurement system technology, the infrared contact temperature measurement method obviously has unparalleled technical advantages. Because of these advantages, infrared external temperature measurement control technology has been widely used in industrial fields in China, such as steel, power, forest engineering, fire protection, petrochemicals, and aerospace [18]. From the perspective of the historical development of infrared optical temperature measurement technology, the rapid development of instruments is mainly concentrated in two main aspects. The first aspect is the continuous development of optical technology. In recent years, the development of infrared optical temperature measurement instrument technology has been extremely rapid. With the continuous development of the cold infrared temperature measuring focal plane, it has advanced rapidly. The second aspect is the continuous development of infrared optical temperature measuring instruments. They are also being rapidly developed along with the continuous development of infrared optical temperature-measuring instrument technology [19,20].

The principle of an infrared thermometer is based on the principle of infrared radiation. Like other technologies, its development has progressed from simple to complex. The earliest infrared temperature measurement equipment could only measure the temperature of a certain point in the field of view, and the accuracy of the temperature measurement was not sufficiently high [21]. With the development of technology, the ability to measure temperature gradually improved; however, temperature measurement devices could not reflect the appearance and shape of the object under study, nor could they be used to determine the surface temperature distribution of the object. The specific reason is that infrared temperature measurement equipment did not enable infrared imaging at the time.

### Features

#### Quick Temperature Measurement

Infrared thermometers respond very quickly to temperature and can be used for real-time and fast-track measurements. Because of this advantage, infrared temperature measurement technology is widely used in the steel and electricity industries, forest fire prevention, and many other aspects.

#### Wide Temperature Measurement Range

Infrared temperature measurement technology has a wider application range than traditional temperature measurement methods. In theory, infrared temperature measurement has no upper limit. In practical applications, infrared thermometers can measure low temperatures of tens of degrees below zero and high temperatures of thousands of degrees. There is no time limit; work can be performed at any time of day or night. Due to the characteristics of infrared technology, the ability to work at night has become a highlight of infrared thermometers. They can be used measure the temperature of microscopic targets. Infrared thermometers are not limited by the distance and can measure the temperature in a short distance or a long distance.

There are many methods of infrared temperature measurement. According to these different methods, current infrared temperature measuring instruments can be divided into two categories: one is infrared temperature measuring equipment based on full-field analysis; the other is infrared temperature measuring system based on point-by-point analysis. The principle of full magnetic field analysis is that the temperature distribution of the entire object is an infrared focal plane array imaging infrared lens, and the temperature distribution of the entire object constitutes the infrared thermal image of the object, and the infrared temperature measurement equipment of the complete field distribution is also called the infrared heat imager [22]. The measurement principle of point-by-point analysis is to focus part of the infrared radiation on an object through an infrared detector, and then according to the object with known surface emissivity, the radiant power of the object is converted into temperature information, and the phase can be easily measured by comparison. This point-by-point analysis system is usually called an infrared thermometer.

IRTI technology did not develop rapidly until the 1950s, and the development of infrared temperature measurement technology has since achieved a fundamental breakthrough [23]. The main function of an infrared thermometer is to measure temperature, and there are three main types: infrared point thermometers, infrared scanners, and infrared thermal television. Objects in nature with temperatures above absolute zero will usually emit a large amount of infrared heat radiation around the temperature measurement image; therefore, the infrared environment in the temperature measurement system may also directly affect the image accuracy of the temperature measurement of the object. This environmental impact factor can be roughly divided into two major parts: the subjective background environmental factor and the objective environmental factor. The background change factor mainly refers to the change difference between the target to be measured by a temperature signal measurement processing system and the surrounding temperature of the measurement background [24,25]. When the difference value is larger, the temperature measurement is more accurate; the natural environmental impact factors are mainly due to the direct impact of environmental factors such as direct solar ultraviolet radiation, direct ground radiation, and wind speed on the accuracy of atmospheric temperature standard measurement results [26]. When the infrared detector detects the target object, it requires the target to be different from the surrounding environment in at least one place for it to be detected by the detector. The greater the difference between the target and the environment, the easier it is to detect; the smaller the difference, the less likely it is to be detected. A comparison is made of the target environment. If the target object is placed in a specific background, then it will be affected by the background. At the same time, as the distance increases, the relative field of view of the same object decreases, which reduces the quality of the output signal and affects the accuracy of the temperature measurement [27].

Using a 2D image to display the 3D distribution of human body temperature takes advantage of the different characteristics of multisensor images and the complementary advantages of multisource images to fuse multisensor images into one image, thereby obtaining a more accurate description of the observed image, scene, or target [28,29]. At present, the most common and widely used image fusion technology is infrared and visible light image fusion. The infrared sensor uses the thermal radiation characteristics of the object itself to “actively” acquire the target information on the image, which has the characteristics of not being affected by the weather or light environment. However, at the same time, due to the limitations of imaging equipment, the spatial resolution and image contrast of infrared images are low, the reflectivity of target details is poor, and the imaging effect does not conform to human visual habits [30]; in contrast, visible light sensors can detect reflections in the scene. Under visible light, the obtained image has higher spatial resolution, clear texture information, and rich image details [31,32]. However, it can be readily disturbed by external factors, and a large amount of scene information will be lost in the case of turbid atmosphere and insufficient light. The fusion of infrared images and visible images can give full play to the advantages of infrared and visible images. The characteristics of these two images are complementary. While maintaining the anti-interference characteristics of infrared images, increasing the spatial details of the image is conducive to improving the detection and reconnaissance capabilities of the system and enhancing the ability of the image system to express the scene [33].

### Infrared Point Thermometry

An infrared point thermometer is a nonimaging infrared thermometer. It can only measure the temperature of a very small area (which can be seen as a point). According to different design principles, infrared point thermometers can be divided into three categories: total radiation thermometers, colorimetric thermometers, and brightness thermometers [34]. Infrared spot thermometers have been available since the early 20th century. The narrow temperature measurement range of these thermometers is not suitable for measuring the temperature of large areas. When it is necessary to measure a large object, it is very inconvenient to obtain multiple measurements of various parts of the object and perform manual scanning. However, these spot thermometers have become a powerful tool in many fields due to their low price and practical features. An infrared scanning instrument, as the name suggests, is an instrument used to detect the linear temperature of an object. The realization principle is generally to detect the temperature of the object through the movement of the object or the cooperation of the atmosphere [35]. Due to the difficulty of realizing infrared scanning instruments and their low commercial value, the application of these instruments is not very common at present. Infrared thermal television is related to infrared point thermometry. It can perform 2D temperature detection on objects, but it does not need to measure multiple points of objects as infrared thermometers do [36]. Its convenience far exceeds that of infrared thermometers. Moreover, its temperature detection does not require a cooling mechanism and can achieve better performance. However, there is a large gap between its technical indicators and infrared cameras. At present, not only single-color thermometers but also two-color thermometers are in use in China. The single-color thermometer can only determine the temperature by the energy of one band, while the two-color thermometer can finally determine the temperature by comparing the radiant energy of two different infrared bands; therefore, this method has strong anti-interference and adaptability [37]. Any object that exists in nature will emit infrared radiation as long as its temperature is above absolute zero. The energy radiation per unit time of the measured object on its surface elements can be expressed as:

T = εσ^4^

Infrared temperature measurement technology is a noncontact temperature measurement technology; it has many advantages compared with contact temperature measurement approaches. However, to reach the infrared detector, the infrared radiation emitted by the target object must pass through the atmosphere, and the atmosphere will absorb infrared light accordingly. At the same time, because of the differences in emissivity of the object, the reflection of the detector, and other factors, the infrared radiation received by the detector is not completely emitted by the target object [38].

In fact, the infrared radiation received by the infrared detector mainly includes the target's own radiation, atmospheric radiation, and environmental reflection [39]. To enable infrared wireless temperature measurement and monitoring system equipment to accurately measure indoor temperature in real time, it is necessary to statistically analyze the data of the main factors that affect the accuracy of the infrared wireless temperature measurement system. Passive detection of the infrared emissivity of a temperature-measuring object is usually a direct influencing factor that directly affects the temperature measurement of infrared rays [40]. The infrared emissivity of an object is usually an important measurement index that is used to characterize the thermal intensity of the measured object on the infrared radiation of its surface. For two objects with the same infrared temperature, the infrared surface temperature emissivity values are different, and the infrared temperature radiation may change; therefore, the infrared temperature radiation received by the infrared radiation detector may also change, which directly affects the intuitive accuracy of the temperature radiation measurement of the object. The influence of the emitted optical power is closely related to many factors, such as the size of the emitting object, the surface temperature, and the wavelength of the light radiated from the object. The power of the surface laser emission is also related to the detection of the coating roughness of the surface of the object shell, the impurities of the chemical compounds and contaminants, and the thickness of the oxide layer of the object [41]. In general, the rougher the laser surface of the same textured object, the higher the value of the laser emissivity of the surface where it is located. In contrast, the smoother the surface object, the lower the surface emissivity. Therefore, it is difficult to measure the emissivity of the object surface very accurately. The atmosphere also contains many polymer gases that can absorb a large amount of infrared light at the same time [42], especially white gas containing water vapor, carbon dioxide, ozone, etc, in the surface layer of the earth's atmosphere, they can also absorb a large amount of infrared light and radiation at the same time, and the concentration of carbon dioxide containing water vapor and hydrogen in the earth's atmosphere is relatively high. In this way, the infrared target detection object mainly refers to the infrared ray radiation gas emitted by the earth. The small molecules that absorb these radiation gases are exposed to the earth's atmosphere, which greatly reduces the infrared radiation activity intensity of the infrared radiation detector; this not only directly affects the infrared temperature measurement accuracy of the sensitive temperature measurement and processing system but also may directly affect the temperature sensitivity of the infrared temperature sensitive measurement and processing system [39]. However, the atmosphere will also attenuate other factors that affect the accuracy of temperature measurement, such as the infrared radiation reflected by the surface of the object, which is much smaller than the infrared radiation intensity of the object itself. If the attenuation of the atmosphere can be fully used, the temperature measurement error can be reduced. Another reason for the attenuation of infrared radiation is the scattering of various gas molecules and suspended particles in the air.

Image fusion technology has important application prospects and substantial application value in both military and civilian fields. Image fusion is a comprehensive process that includes image acquisition, image transmission and image signal processing. Image acquisition and transmission require the functions of the image sensor and its related hardware circuits [43]. Image signal processing relies on software algorithms running on hardware platforms. The entire image fusion system is a complex system combining software and hardware. According to their working modes, infrared detectors can be divided into two categories: cooled infrared focal plane detectors and uncooled infrared focal plane detectors. The advantages of cooled infrared focal plane detectors are their high sensitivity and long detection distance; however, their use conditions are harsh and expensive [44]. At present, only a few of these detectors are used in high-end military equipment; uncooled infrared focal plane detectors can work directly at room temperature, are small in size, consume low power, and demonstrate fast startup; moreover, the price is only a small part of the cooling type. Infrared radiation is very common in nature; however, because the wavelength of infrared radiation is invisible to the human eye, people cannot observe it. Therefore, people began to attempt to design infrared equipment, such as IRTI, to replace the human eye, extend the visual range of the human eye from visible light to the infrared band, and directly obtain the shape characteristics and temperature distribution of objects that cannot be observed in the visible light range. When the internal temperature of the object is in an unbalanced state, a heat transfer process occurs [45]. In this process, discontinuous measurements of the objects will affect or hinder the normal transmission and distribution of heat and the temperature differences of different areas on the surface of the object will then be measured, resulting in different temperature distribution patterns, that is, “hot spot” and “cold spot” thermal images. Because the temperature distribution can indirectly reflect the physical characteristics of the measured object, according to the basic law of infrared radiation, IRTI detection technology is processed by charge-coupled devices in infrared equipment, such as infrared cameras [46]. The infrared radiation signal containing the target defect information is converted into a visible light thermal image, and the defect information of the measured object is extracted in combination with the corresponding data processing method. At present, in the field of IRTI detection, the topics studied by researchers mainly include three aspects. The first aspect is the use of different excitation methods; the second is expansion of the scope of application; and the third is the optimization of thermal image processing. In the early research of thermal image data processing methods, the data processing method based on pulsed eddy current thermal imaging detection technology is generally to select the best thermal image to extract defect features and then complete the defect identification [47].

### Acute Upper Respiratory Tract Infection

Upper respiratory tract infection is a common and frequent disease of the respiratory system. Its incidence is very high, it can spread in a small area, and it can even cause pandemics [48]. It can cause myocarditis, acute nephritis, encephalitis, and even respiratory distress syndrome. In recent years, the harm caused by severe acute respiratory syndrome (SARS) and H1N1 influenza to humans is still vivid. China has a high rate of acute upper respiratory tract infections.

IgG has antiviral, antibacterial, and complement fixation functions, and it participates in mucosal immunity through placenta and IgA [49]. The decline of IgG reduces the body's antibacterial and antiviral ability, which can easily lead to repeated respiratory infections. Increasing numbers of patients with repeated respiratory tract infections have low specific pneumococcal antibody levels or are unable to respond adequately to pneumococcal vaccines or natural infections. This may explain why many patients do not experience a decline in total immunoglobulin or immunoglobulin subclasses although immunodeficiency of pneumococcal and other capsular bacteria occurs. Any decrease in cellular immune function can cause respiratory infections. IL-2 can promote the transformation of T cells from the thymus and spleen to cytotoxic cells, thereby enhancing granulocyte antibody–dependent cell-mediated cytotoxicity [50]. At the same time, IL-2 has been found to have antiviral effects and protect adults from Epstein-Barr virus infection. Therefore, in the treatment of respiratory tract infections, in addition to active antibiotic treatment, recombinant cytokines or cytokine antagonists should also be provided with an appropriate microenvironment to regulate the level of cytokines, change the immune response in the body, and achieve the purpose of treatment.

Smokers are twice as likely to have acute upper respiratory tract infections as nonsmokers [51]. Serum bilirubin levels may be an influencing factor. People with low levels of serum bilirubin are more likely to develop symptoms of acute upper respiratory tract infection.

Exposure of children to smoke can increase their incidence of lower respiratory tract infections (such as bronchitis and pneumonia) and can induce new asthma. A normal chemical composition in the atmosphere is a necessary condition to ensure human health. With the development of industrial production and transportation, the use of coal, oil and other energy sources has increased, and a large number of harmful substances are being scattered in the air [52]. Air pollution has become one of the main factors endangering human health [53]. Acquired risk factors include vitamins, trace element deficiency, diabetes, and other related factors. It was found that the content of vitamins A, E, and B and of carotene in the serum of children with repeated respiratory infections was significantly reduced. Vitamin A plays an important role in enhancing human immune function. In the absence of vitamin A, T cells may undergo nonspecific changes, and T cell proliferation may also be impaired. T cells play an irreplaceable role in various immune inflammatory responses. Calcium, magnesium, and other elements play an important role in regulating enzyme function, cell activity and body immunity. When the body lacks calcium, chronic respiratory diseases easily occur and are readily aggravated. Magnesium is involved in regulating immune function and increasing the body's resistance [54]. Diabetic patients are also susceptible to infection, and the infection is serious and difficult to control in these patients.

### Aim of This Study

In this paper, we use experimental research methods to understand the impact of IRTI through qualitative and quantitative studies and a comparative exploration before and after the application of this technology; through theoretical analysis and experimental exploration, the role of IRTI in the diagnosis of acute upper respiratory tract infection can be elucidated. Data were recorded, sorted, calculated, plotted, and analyzed for processing, statistical analysis was performed on the IRTI data set, and empirical analysis of infrared expression using IRTI technology was performed for patients with acute upper respiratory tract infection. The location and characteristics, combined with the effective data, summarize and analyze the important role of IRTI technology in clearly expressing the quantification of temperature. The results show that with the method described in this paper, superior research results are obtained.

## Methods

### Experimental Data

From December 2018 to February 2019, 154 patients with acute upper respiratory infection were randomly selected in the emergency department of the First Affiliated Hospital of Guangzhou Medical University. The patients included 73 men and 81 women. According to fever, the subjects were divided into two groups: fever type and nonfever type. If the patient’s axillary temperature reached the fever threshold (≥37.1 ºC), they were classified as fever type. Among the 154 participants, 76 were in the fever group; the ratio of men to women was 40/36, the age range was 14-81 years, and the average age was 37.70 years (SD 18.54); 78 patients were in the nonfever group, the ratio of men to women was 33/45, the age range was 14-87 years, and the average age was 38.76 years (SD 17.19). We also established a control group with 40 patients; the male to female ratio in this group was 19/21, the age range was 19-80 years, and the average age was 39.20 years (SD 19). Patients were excluded if they had high metabolism specific to head and face tissue (eg, tumors, infections), abnormally low metabolism (eg, benign nodules), or abnormal blood flow (eg, hemangioma).

### Questionnaire

A questionnaire was developed according to the design of the study, including questions related to general personal and family information, previous medical history (tuberculosis, chronic bronchitis, asthma, hypertension, diabetes, etc), smoking history, drinking history, personal history, and the presence of acute upper respiratory tract infection within 1 year after physical examination.

### Statistical Processing

SPSS 20.0 data analysis software (IBM Corporation) was used for single factor logistic regression analysis. The dependent variable (defined: l, not defined: 0) was whether an acute upper respiratory tract infection occurred, and each study factor used a single factor logistic regression equation as the independent variable. We selected variables with *P*<.05 as statistically significant, and a multiple unconditional logistic regression model was then fitted to the selected variables.

### Experimental Method

An ATIR-M301B far-infrared thermal imager (China Chongqing Weilian Technology Co Ltd) and uncooled focal plane digital thermal imaging technology were used; the temperature resolution was 0.05 ºC, and the spatial resolution was 2 mrad. In the examination room without obvious air convection, the room temperature was controlled at 23 ±2 ºC. Before the inspection, the test site was exposed until the signs were stable and the skin temperature was appropriate. When the equilibrium temperature of the detection environment had been established for 5 to 15 minutes, the far-infrared thermal imager was used to collect far-infrared thermal images of the participant’s head and face. The far-infrared thermal imager can scan and collect the infrared thermal energy emitted by the human body, and through computer analysis and processing, it can express different temperatures with different colors; therefore, it is an intuitive far-infrared thermal imaging system. By observing the images, it is possible to accurately analyze changes in body surface temperature due to changes in the nervous system and analyze diseases. Diseases can be discovered at an early stage, and the changes and results of the disease can be observed in a timely fashion. Far-infrared thermal imaging has the characteristics of safety, convenience, and low cost. The images be checked repeatedly to dynamically observe changes in a patient’s condition. The ambient temperature was 22 to 24 ºC, with no heat source interference, humidity of 50% to 60%, no strong light, and no wind. The instrument complies with national standards (GB/*t* 1965-2005). The instrument was equipped with a 320 × 240 uncooled focal plane infrared collection lens. The spectral response was 8 to 14 μm, the spatial resolution (instantaneous field of view) was 1.3 mrad, the temperature resolution was 0.05 ºC, and the acquisition speed was 30 frames/s. The thermal structure of the human body could be displayed objectively and digitally in the form of pseudocolor heat. All subjects were prepared in accordance with IRTI testing specifications before the test. During the test, the participant assumed the correct sitting posture and exposed the examination site, 2 meters away from the infrared lens, 1 faced vertically, 2 face up, and so on. Focusing, temperature calibration, image acquisition, storage, image processing, etc, were performed according to reference [55].

### Qualitative Index of Infrared Thermal Image Evaluation

We observed the expression and distribution characteristics of the infrared thermal images. Different color scales represented different temperatures (white: super high temperature zone; red: high temperature zone; pink: hot zone; yellow: warm zone; green: cool zone; blue: low temperature zone; black: ultra-low temperature zone). Changes in color scale were compared between groups.

### Quantitative Index of Infrared Thermal Image Evaluation

The average temperature corresponding to the extracted temperatures was quantitatively compared for the patients with upper acute respiratory infection with and without fever and the control group without fever on the left and right nasal areas and the left and right sides of the pharynx. For statistical processing, the data were extracted from the corresponding part of the infrared software system, and SPSS 20.0 was used to organize the data [56]. The measurement data were expressed as mean (standard deviation). We compared the average temperatures using analysis of variance.

Ethical Approval and Consent to Participate The experimental protocol was established according to the ethical guidelines of the Helsinki Declaration and was approved by the Human Ethics Committee of Department of Pediatric Surgery, General Hospital Campus, the First Affiliated Hospital of Guangzhou Medical University. Written informed consent was obtained from individual participants or their guardians.

## Results

### Infrared Thermal Image Analysis

**Figure 1:**
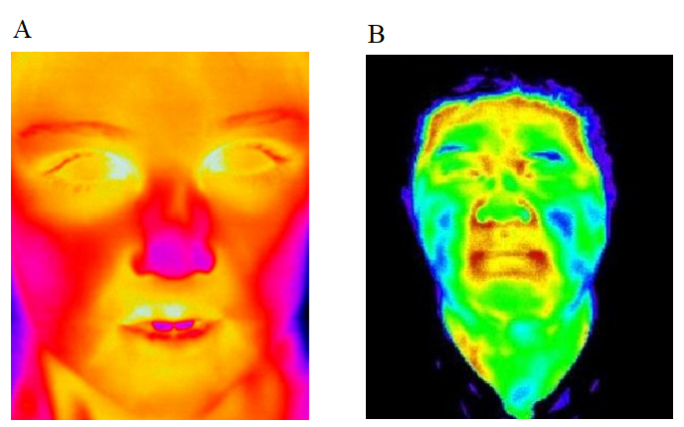
Infrared thermograms of a patient in the fever group (left) and a patient in the nonfever group (right). Infrared thermal image distribution characteristics: white: ultra-high heat area; red: high heat area; pink: hot area; yellow: warm area; green: cool area; blue: cold area; black: ultra-cold area.

According to the statistical analysis of the data, as shown in the examples in Figure 1, the infrared thermograms of the patients in the nonfever group mainly showed “low temperature” blue or green color, the infrared expressions on the left and right sides have good symmetry, and the average temperature difference does not exceed 1 °C. The expression is similar, and the corresponding body surface area is a focal or large-scale focal or diffuse pink hot zone or red hot zone; this effect is more obvious in the fever group than in the nonfever group. Infrared temperature measurement technology avoids direct contact between the thermometer and the object and does not affect the temperature field distribution of the object; this can ensure high temperature measurement accuracy. A high-performance infrared thermometer can distinguish a temperature difference of 0.01 ºC.

### Quantitative Temperature Distribution and Comparison

According to the statistical analysis of the data, as shown in Table 1, the average temperature of the infrared thermal image of the corresponding body surface area of the nasal cavity and larynx in the fever group is higher than that in the nonfever group; however, the average body temperature of both groups is higher than that of the normal control group (*P*=.003).

**Table 1:** Quantitative distribution of temperature of the patients in the fever, nonfever, and control groups (N=194).

Group	n	Temperature (ºC), mean (SD)				
		Nasal region			Throat region	
		Left	Right	Left		Right
Fever	76	36.78 (0.67)	36.85 (0.72)	36.34 (0.66)		36.40 (0.68)
Nonfever	78	34.54 (0.47)	34.63 (0.49)	34.28 (0.53)		34.25 (0.56)
Control	40	33.28 (0.42)	33.37 (0.47)	33.09 (0.49)		33.14 (0.77)

### Analysis of Acute Upper Respiratory Tract Infection

#### Gender Analysis

According to the statistical analysis of the data, as shown in Table 2, the incidence was 24.8% (82/331) for male patients and 13.9% (46/331) for female patients. There was a significant difference between men and women (*P*<.001).

**Table 2:** Analysis of incidence of respiratory infection by gender (n=331).

Gender	Acute upper respiratory tract infections, n	Nonrespiratory infections, n	Incidence rate (%)
Female	46	109	13.9
Male	82	94	24.8

#### Analysis of Related Factors

**Figure 2:**
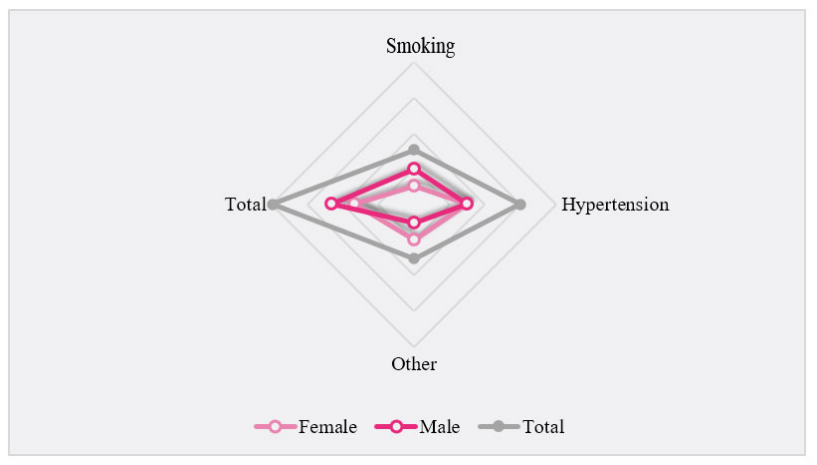
Prevalence of relevant factors among male patients, female patients, and all patients.

According to the statistical analysis of the data, shown in Figure 2, 128 cases of acute upper respiratory tract infection were observed in our study; the prevalence of upper respiratory tract infection by sex was 64.1% (82/128) males and 35.9% (46/128) females, while 68% (87/128) were smokers. It can be seen that smokers were more likely to have acute upper respiratory tract infection than nonsmokers (*P*<.001). At the same time, there was also a significant difference between men and women (*P*<.001). Of these patients, 57.8% (74/128) had hypertension. It can be seen that the incidence of acute upper respiratory tract infection in patients with hypertension was higher than that in patients without hypertension (*P*<.001). At the same time, there was no significant difference in the proportion of men and women with hypertension.

## Discussion

### Principal Findings

We found that that smokers are more likely to have acute upper respiratory tract infection than nonsmokers (*P*<.05), while men had a statistically higher prevalence of acute upper respiratory tract infection than women (*P*<.05). It is known that men smoke more than women [57]. Smoking is a risk factor for respiratory infection. First, the harmful substances in tobacco will destroy the immune monitoring cells in the airway (ie, phagocytes); thus, their phagocytic capacity and lethality will be reduced. Secondly, smoking can stimulate the proliferation of goblet cells; these cells secrete large amounts of mucus and fewer antibodies, which is conducive to the growth of bacteria. At the same time, smoking will destroy the cilia of the ciliated columnar epithelial cells of the respiratory tract, render them shorter, irregular, or incomplete, and prevent the smooth discharge of sputum [58].

Smoking can also directly stimulate the airway, cause airway spasms, and affect sputum drainage. The above adverse factors can weaken the defense ability of the respiratory tract, and the respiratory tract infection causes the damaged ciliated columnar epithelial cells to gradually develop into squamous epithelium. On the one hand, this weakens the purification ability of the airway; on the other hand, it also carries the risk of cancer. As an independent and serious risk factor of respiratory tract infection, smoking has attracted increasing attention in recent years. The most common respiratory diseases associated with smoking are lung cancer, bronchitis, and emphysema [59,60].

### Conclusions

IRTI can provide an objective and quantifiable reference for the visual diagnosis of people with acute upper respiratory infection, and it can be used as one of the effective indicators of clinical diagnosis. There are obvious abnormal expressions of infrared thermography in the corresponding parts of the body surface.

The pathological features of acute sinusitis and acute pharyngitis are inflammatory changes with vascular exudation as the central link, and the local manifestation is elevated body temperature.

Because IRTI is highly sensitive to vascular disease or inflammation, infrared detection can show that the average temperature of the nasal cavity area and the middle neck throat area of the corresponding surface of acute sinusitis and acute pharyngitis is significantly higher, showing typical abnormal thermal images.

## Abbreviations

IRTI: infrared thermal imaging
SARS: severe acute respiratory syndrome
